# Predictive modelling of clinically significant depressive symptoms after coronary artery bypass graft surgery: protocol for a multicentre observational study in two Swiss hospitals (the PsyCor study)

**DOI:** 10.1136/bmjopen-2025-108061

**Published:** 2025-09-05

**Authors:** Asimina Lazaridou, Sinthujan Sivakumar, Hector Rodriguez Cetina Biefer, Sonja Weilenmann, Mary Princip, Claudia Zuccarella-Hackl, Frederike H Petzschner, Jakob Heinzle, Klaas E Stephan, Omer Dzemali, Roland von Känel

**Affiliations:** 1 Brigham and Women’s Hospital, Chestnut Hill, Massachusetts, USA; 2 Department of Consultation-Liaison Psychiatry and Psychosomatic Medicine, University Hospital Zurich, University of Zurich, Zurich, Switzerland; 3 Department for Cardiac Surgery, University Hospital Zurich, University of Zurich, Zurich, Switzerland; 4 Department for Cardiac Surgery, City Hospital Zurich - Triemli, Zurich, Switzerland; 5 Centre for Experimental and Translational Cardiology, University of Zurich, Zurich, Switzerland; 6 Cognitive and Psychological Sciences and Robert J. and Nancy D. Carney Institute for Brain Science, Brown University, Providence, Rhode Island, USA; 7 Translational Neuromodeling Unit, Institute for Biomedical Engineering, University of Zurich and Swiss Federal Institute of Technology Zurich, Zurich, Switzerland; 8 Max Planck Institute for Metabolism Research, Cologne, Germany

**Keywords:** Coronary heart disease, Machine Learning, Depression & mood disorders, Anxiety disorders

## Abstract

**Abstract:**

**Introduction:**

Coronary artery bypass grafting (CABG) remains one of the most commonly performed cardiac surgeries worldwide. Despite surgical advancements, a significant proportion of patients experience psychological distress following surgery, with depression being particularly common. Current evidence regarding the effectiveness of preoperative psychological interventions in improving postoperative mental health outcomes remains inconclusive. There is a critical need for predictive models that can identify patients at risk of developing clinically significant depressive symptoms (CSDSs) and related psychological conditions after CABG. This multicentre observational study aims to develop and validate prognostic models for predicting CSDSs and other psychological outcomes, including anxiety, post-traumatic stress symptoms and quality of life, 6 weeks after elective CABG surgery.

**Methods and analysis:**

The study will recruit 300 adult patients undergoing elective CABG (with or without valve intervention) across two Swiss hospitals. Data collected will include demographic, clinical, psychometric, inflammation-related and interoceptive variables. A training set (n=200) will be used to develop predictive models using machine learning, while a held-out test set (n=100) will be used for model validation. The primary outcome prediction will focus on CSDSs, assessed using the Patient Health Questionnaire-9 (PHQ-9), with analyses conducted both categorically (PHQ-9 total score ≥10) and continuously as complementary approaches. Secondary models will address anxiety, using the General Anxiety Disorder Scale-7, post-traumatic stress, using the post-traumatic stress disorder checklist for Diagnostic and Statistical Manual of Mental Disorders-5 and health-related quality of life, using the 12-item Short Form Survey. A simplified ‘light solution’ model with fewer predictors will also be developed for broader applicability. This study will address an important gap in perioperative mental healthcare by identifying key predictors of psychological morbidity following CABG, particularly CSDSs. The resulting models may inform future screening and preventive strategies and improve postsurgical outcomes through early identification and intervention in high-risk individuals.

**Ethics and dissemination:**

The responsible ethics committee has reviewed and approved this project (Kantonale Ethikkommission Zürich, BASEC number: 2023-02040). The study minimises participant burden by integrating brief validated instruments and limiting psychiatric interviews to relevant outcomes, while ensuring ethical safeguards and respect for participant rights (including written consent). Results will be shared through peer-reviewed publications, conference presentations and stakeholder meetings involving clinicians and mental health professionals. Findings will also be communicated to participating centres and patient communities in accessible formats.

**STRENGTHS AND LIMITATIONS OF THIS STUDY**

- This is a multicentre study aiming to develop and validate machine learning-based prediction models for psychological outcomes following coronary artery bypass grafting, including depression, anxiety, post-traumatic stress disorder symptoms and quality of life.
- The study combines psychometric, clinical, inflammatory and interoceptive markers, enabling a multimodal approach to risk prediction.
- Use of both categorical and continuous outcome measures increases model robustness and clinical relevance.
- A separate validation cohort will be used to assess model generalisability and reduce the risk of overfitting.
- As an observational study, causal inferences cannot be drawn, and potential site-specific differences between the two hospitals may limit external validity.

## Introduction

 Coronary heart disease (CHD) continues to be the leading cause of death worldwide across all economic settings, despite a gradual decline in mortality rates. This decline is largely attributed to improvements in healthcare infrastructure and advances in the management of cardiovascular risk factors.^1^ For patients with stable CHD who are on guideline-recommended conservative therapy, the continuation of ischaemic symptoms despite medical treatment and the need for improved prognosis are triggers for considering invasive interventions such as percutaneous coronary intervention (PCI) or coronary artery bypass grafting (CABG).^2^ These procedures are intended to restore adequate blood flow to obstructed coronary arteries. The choice between PCI and CABG is guided by a thorough evaluation of the risk-benefit profile for each patient. In more complex cases of CHD, such as those involving an unprotected left main coronary artery or de novo three-vessel disease, CABG is typically the preferred strategy for achieving complete myocardial revascularisation. In certain cases, CABG may also be combined with valve interventions, particularly in patients with a primary indication for aortic or mitral valve surgery and concurrent coronary artery stenosis exceeding 70%, or in patients with severe aortic stenosis undergoing CABG. Achieving survival benefits requires complete revascularisation, by PCI or CABG, of all epicardial vessels ≥1.5 mm in diameter with ≥50% luminal stenosis visible in at least one angiographic view.^3^

CABG remains the most commonly performed cardiac surgery worldwide, with approximately 200 000 isolated procedures annually in the USA and an average incidence rate of 62 per 100 000 inhabitants in Western European countries.^4^ However, despite its widespread use, postoperative quality of life (QoL) may be overestimated, particularly in older, high-risk patients and those with already diminished QoL prior to surgery.^5^

One major factor influencing postoperative outcomes and perceived QoL is depression. Depression is a debilitating mental health disorder characterised by affective symptoms such as low mood and loss of interest, cognitive difficulties including impaired concentration, fatigue and somatic complaints, sleep disturbances and appetite changes. It is also one of the most common comorbidities in patients with chronic medical conditions, including CHD.^6^ Depending on measurement tools, the prevalence of depression pre-CABG ranges from 19% to 37%.^7^ Adverse health behaviours and biological mechanisms such as autonomic, neuroendocrine and endothelial dysfunction, low-grade inflammation and coagulation activation may partially explain this link.^8 9^ Depression (ie, both major and minor depression) is arguably the most reliably identified psychosocial risk factor of incident CHD^10 11^ and of poor prognosis after an acute coronary syndrome (ACS).^12^ In patients with CABG, clinically significant depressive symptoms (CSDSs) and anxiety symptoms frequently co-occur and are associated with poor prognosis after CABG. Specifically, meta-analyses show an association of perioperative depression with a significant 1.5-fold increased late all-cause mortality risk.^13^,^15^

In addition, the prevalence of perioperative anxiety was estimated to be 22% in patients undergoing CABG with or without combined valve intervention.^15^

A recent meta-analysis demonstrated that perioperative patient information can reduce postoperative anxiety, but not depression, pain and hospital length of stay. Also, a pre-surgery intervention optimising expectations about the course and outcome after cardiac surgery resulted in less disability and better QoL 6 months after CABG, although not in decreased depressive and general anxiety symptoms, compared with standard medical care or a support/advice control intervention.^16^ A secondary moderator analysis of this trial suggested that for patients with elevated depressive symptoms before surgery, both optimising expectations and providing emotional support pre-surgery decreased depressive symptoms at 6 months after CABG more than standard medical treatment.^17^ In another study, brief cognitive behavioural therapy during hospitalisation in patients undergoing transcatheter aortic valve replacement at high or prohibitive surgical risk showed no benefit after 1 month in reducing symptoms of depression and anxiety compared with treatment as usual.^18^ This suggests that biological mechanisms may underlie postsurgical affective symptoms beyond psychological interventions. Inflammation plays a key role in depressive symptoms across disorders with an inflammatory component.^6^ Both low-grade systemic chronic inflammation and acute inflammatory responses have been linked to CSDSs in patients with CHD, including those who have undergone CABG. Elevated high-sensitivity C reactive protein (hsCRP) levels measured 3 days before CABG were associated with CSDSs at 6 months postsurgery (18% of patients), independent of demographic and clinical variables.^19^ Similarly, elevated interferon (IFN)-γ 1–3 days post-CABG were associated with CSDSs at 12 months (20% of patients), comparable to pre-CABG depressive symptom levels.^20^

Other markers, such as white cell count during ACS, were linked to elevated depressive symptoms at 3 weeks and 6 months.^21^ Interleukin-6 (IL-6) and IL-8, measured 2–14 weeks post-ACS, were associated with mood disorders at baseline and 1 year follow-up.^22^ Higher hsCRP levels at 3 years were observed in patients with CHD who developed CSDSs.^23^ Additionally, acute-phase inflammation and coagulation markers such as fibrinogen and D-dimer predicted depressive symptoms in healthy individuals^24^ and patients with ACS.^25^

Confounders such as medications, physical activity and cardiac function influence this relationship. For instance, physical activity pre-CABG was inversely related to CRP levels.^26^ Greater inflammatory response (IL-6, IL-8) post-CABG was observed in patients with low ejection fraction.^25^ Statin use at discharge was associated with reduced CSDSs risk at 3 and 9 months.^27^

The inflammation hypothesis of depression suggests that cytokines alter tryptophan metabolism, leading to neurotoxic metabolites affecting serotonin, dopamine and glutamate systems.^28^ Meta-analyses have demonstrated that low-grade inflammation (CRP >1–3 mg/L) raises depression risk 1.5-fold.^29^ Older adults show elevated IL-6 levels; higher baseline IL-6 and CRP predicted depressive symptoms, independent of other risk factors.^30^ Longitudinal studies have confirmed bidirectional associations between CRP, IL-6 and depression.^31^

In addition, peripheral hsCRP levels strongly correlate with cerebrospinal fluid inflammatory markers, making hsCRP a proxy for central inflammation.^32^ CRP, IL-1β, tumour necrosis factor (TNF) and IL-6 are associated with anhedonia and motivational deficits.^33^ Anti-inflammatory treatments (anti-TNF and anti-IL-6) significantly reduce depressive symptoms.^34^,^36^ Antidepressants may lower IL-6 and TNF-α,^37 38^ but non-responders have elevated IL-8 and persistent TNF-α.^39^ Higher CRP and IL-8 levels are also linked to antidepressant resistance.^40^ Nonetheless, trials of agents such as statins and aspirin have shown mixed results.^41 42^

Collectively, the effectiveness of psychological interventions delivered before cardiac surgery with the aim to improve psychological recovery after surgery remains inconclusive. Therefore, the possibility of exerting a preventive influence on CSDSs post-CABG, based on a prediction model, is particularly important, as depression is highly prevalent, considerably persistent and associated with adverse outcomes in patients who undergo CABG. The potential role of inflammation, a biological mechanism implicated in the onset and persistence of depressive symptoms, has not yet been sufficiently integrated into models for CSDSs. Similarly, interoception, the perception of internal bodily states including cardiac signals,^43 44^ may be impaired in depression and could contribute to individual differences in psychological recovery after surgery.^45^ The primary aim of this study is to develop and evaluate prognostic prediction models of CSDSs and other indicators of psychological distress, namely, anxiety, post-traumatic stress and health-related QoL at 6 weeks after elective CABG surgery.

## Methods and analysis

### Study setting

This will be a multicentre observational study involving three subcentres. The Department of Consultation-Liaison Psychiatry and Psychosomatic Medicine at the University Hospital Zurich (USZ) is responsible for the psychiatric components of this study. The Department of Cardiac Surgery at the University Hospital Zurich and Stadtspital Zurich Triemli are responsible for the medical and cardiac-related tasks. It is important to emphasise that the two study sites are spatially and organisationally distinct. To minimise the risk of information leakage, which could compromise the validity of predictive models, study personnel at the respective sites will be non-overlapping. For psychiatric responsibilities, the USZ will assemble a team with a psychological background, including research assistants. The medical and cardiac surgery components will be managed by the respective cardiac surgery departments at the USZ and Triemli. Each subcentre will be responsible for the training and qualification review of its designated study personnel: psychiatric and psychological training will be overseen by the USZ, while the medical teams at the USZ and Triemli will manage training for cardiac and medical tasks.

### Overview of study design

We will develop a prognostic prediction model for CSDSs, assessed using the widely used Patient Health Questionnaire (PHQ)-9, at 6 weeks post-CABG, using demographic, health behaviour, psychometric, medical, inflammation and (cardiac) interoception data. Data analysis will be performed using machine learning (ML) algorithms. We will recruit a total of 300 patients from cardiac surgery departments in two different hospitals. Dropouts due to a loss to follow-up will be replaced by recruiting additional patients until n=300 is reached. This final sample will consist of 200 patients recruited at University Hospital Zurich for the training dataset which will serve to construct candidate predictive models of CSDSs. Another 100 patients will be recruited at Stadtspital Zurich Triemli; these data will constitute the test dataset for validating the final model. Determining sample sizes for out-of-sample prediction studies is challenging because, in contrast to power analyses for within-sample analyses, there are no standard analytical procedures. For this reason, the planned sample size of our study is motivated by a recent simulation study to pinpoint sample size requirements for multivariate models applying ML to predict between-patient differences in treatment of major depression. This study focused on the question of what sample size was needed to identify ‘prescriptive predictors’ of treatment outcome, that is, predictor variables showing an interaction with treatment type in clinical trials with two active treatment arms. According to this simulation study, at least 300 patients per treatment arm are recommended to detect clinically significant underlying marginal improvements in treatment response. This study emphasised that identifying such prescriptive predictors posed a much greater challenge in terms of statistical power than finding ‘prognostic predictors’, that is, variables showing a main effect, predicting treatment response irrespective of treatment type. (In other words, the differential statistical power for identifying prognostic versus prescriptive predictors corresponds to the well-known difference in power between testing for main effects and interactions.) In our case, we deal with a single patient group and a single treatment type; our problem thus corresponds to finding prognostic predictors. Training and test datasets will remain strictly separated throughout the entire study; this extends to the level of data-acquiring study personnel which will be non-overlapping for the two hospitals. To avoid model overfitting, a nested cross-validation approach will be used for the training dataset. One analysis approach will perform a binary prediction of absence/presence of postoperative CSDSs, using a PHQ-9 cut-off score ≥10, as proposed for depression screening in patients with coronary artery disease. A second approach will adopt a continuous perspective, using regression to predict PHQ-9 scores. In addition to the comprehensive prediction model, a ‘light solution’ model will also be calculated and evaluated with candidate predictors that are generally available in all cardiac surgery centres, in order to increase generalisability and facilitate translation into practice. The data will also be used to develop predictive models for CABG-induced anxiety symptoms, post-traumatic stress symptoms and QoL.

### Specific aims

*Aim 1*: The primary aim of this project is to establish a prognostic predictor model for post-CABG CSDSs (as assessed by the PHQ-9 questionnaire).

*Hypothesis 1*: We hypothesised that in patients undergoing elective CABG, the collected data allow for establishing a prediction model of the absence versus presence of CSDSs (PHQ-9 score ≥10) at 6 weeks post-CABG, with a balanced accuracy that is significantly above chance (assessed by permutation testing).

*Aim 2*: With the data collected to achieve the primary aim, additional prognostic prediction models will be specified for CSDSs (PHQ-9 continuous score) and further measures of poor psychological well-being post-CABG, namely increased anxiety, CABG-induced post-traumatic stress and reduced QoL. A ‘light solution’ model of post-CABG CSDSs will be established with candidate predictors available in all cardiac surgery centres.

*Hypothesis 2*: We hypothesise that in patients undergoing elective CABG, the collected data enable us to establish a prediction model of the continuous PHQ-9 score of patients at 6 weeks post-CABG, with a prediction accuracy that is significantly above chance (assessed by permutation testing). We further hypothesise that the collected data allow for establishing prediction models of increased anxiety (General Anxiety Disorder Scale-7 (GAD-7) score), increased CABG surgery-induced post-traumatic stress (post-traumatic stress disorder checklist for Diagnostic and Statistical Manual of Mental Disorders-5 (PCL-5) score) and poor QoL (12-item Short Form Survey (SF-12) mental and physical health component scores), all at 6 weeks post-CABG.

### Participant selection and procedures

The collection and description of data will adhere to the guidelines for Strengthening the Reporting of Observational Studies in Epidemiology on the one hand and the Transparent Reporting of a Multivariable Prediction Model for Individual Prognosis or Diagnosis (TRIPOD) Statement. The TRIPOD Statement provides a 22-item checklist to improve the reporting of studies developing, validating or updating a prediction model, whether for diagnostic or prognostic purposes.^46^ Based on a literature review of methodological conduct^47^ and completeness of reporting^48^ of ML-derived prediction models, the TRIPOD-artificial intelligence (AI) extension to the original TRIPOD statement has now been published.^49^ This updated guideline provides specific recommendations for the reporting of clinical prediction model studies that apply ML techniques. Accordingly, the TRIPOD-AI checklist will be applied to guide the analysis and reporting of our study results.

### Recruitment, screening, informed consent and questionnaires

**Figure 1:**
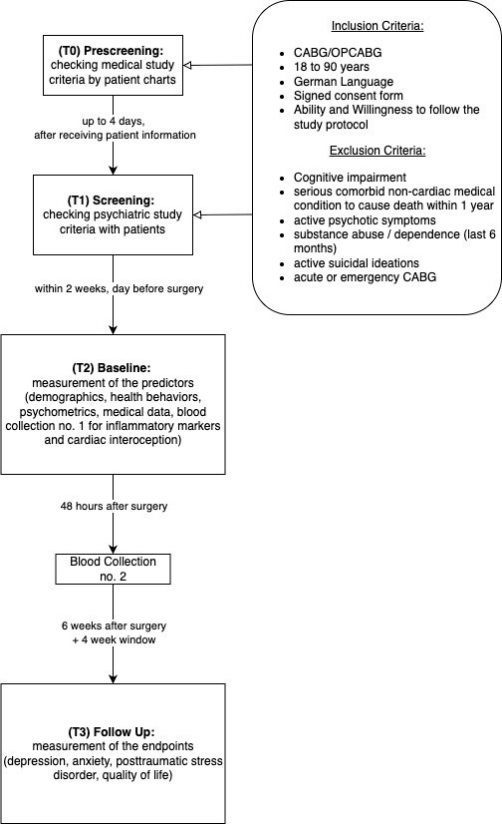
Study flowchart. CABG, coronary artery bypass grafting, OPCABG, Off-Pump Coronary Artery Bypass Grafting.

The cardiac surgeon or study staff of the cardiac surgery department on site (USZ, Triemli) will inquire whether consecutive patients between the ages of 18 and 90, seeking consultation at the University Hospital Zurich (for the training dataset) or Stadtspital Zurich Triemli (for the test dataset) in the cardiac surgery outpatient clinic, and who meet the indication for elective Off-Pump Coronary Artery Bypass Grafting (OPCABG) or CABG, with or without valve intervention, can envision participating in this study (Prescreening, T0). Medical exclusion criteria are taken into account, such as the presence of a serious non-cardiac comorbid condition likely to result in death within 1 year. If the patient declares interest, the detailed study/patient information and the informed consent form (ICF) will be handed to reconsider possible participation, at the latest 1 day before the surgery. Furthermore, permission is obtained to forward the contact details to the main trial centre, allowing them to contact potential participants. The permission is recorded in writing by the trial centre. The patient will be informed that a member of the study team will contact them by phone or on site within the same or the next few days to provide more detailed information about the background and objectives of the study, to answer any further questions about the study and to clarify whether the patient would like to participate or not. Patients will be informed verbally that their participation in the project is voluntary and that they can discontinue their participation in the study at any time. If the criteria for study participation are met, the questionnaires (demographics, health behaviours, some additional questions about the psychiatric history and treatment) and the Mini International Neuropsychiatric Interview (MINI) will be collected by telephone or on site and entered into the electronic case report form (eCRF). This approach reduces the burden of completing questionnaires 1 day before surgery, while increasing the likelihood of obtaining complete data and minimising the risk of missing values. On the day of hospital admission, which is the day before CABG surgery, eligible patients will have another opportunity to ask the USZ PSY study team questions before signing the ICF. Current medication will be inquired and verified from patient charts after the ICF has been signed. Further study-specific interviews, questionnaires (eg, medical and psychological), and examinations (eg, blood samples, body mass index, blood pressure, pulse, waist-hip ratio, heartbeat counting task (HBCT), ECG) are only to be conducted after written informed consent has been obtained, with one exception. Since the blood sample is collected as part of the routine procedure by the nursing staff, an additional tube may be drawn for research purposes before written consent is obtained, provided that oral consent has been given. In such cases, this is specifically documented in writing. The study team will make every effort to ensure that this remains an exception. Participants will receive a copy of the signed consent form. If the consent form is not signed, all patient data entered in the eCRF, collected on paper before or during screening, and blood samples will be deleted or destroyed prior to data analysis. This initial assessment with the patient will take approximately 45 min (see figure 1).

### Eligibility criteria

#### Inclusion criteria

- Elective OPCABG or CABG, either isolated or combined with valve intervention.
- Men and women, aged between 18 and 90 years.
- Sufficient knowledge of German language in reading and understanding.
- Written consent form.
- Ability and willingness to follow the study protocol.

#### Exclusion criteria

- Cognitive impairment according to a score of ≤7 (maximum score=9) on a modified version of a short version of the Mini-Mental State Examination^50^ and the Brief Interview for Mental Status.^51^
- Any serious comorbid non-cardiac medical condition likely to cause death within 1 year (eg, metastatic cancer; will be checked by medical staff and/or verified with the patient).
- Active psychotic symptoms (assessed using two items from the Youth Psychosis At Risk Questionnaire),^52^ substance abuse and/or dependence within the past 6 months (assessed with a single-item questionnaire)^53^ and/or active suicidal ideation (assessed with a single item from the MINI, see below).
- Unscheduled (ie, acute or emergency) CABG.

### Outcomes and screening assessments

#### Mental health variables and psychometric assessment

##### Psychiatric history and diagnoses

The MINI will be used to evaluate the presence of psychiatric disorders according to the Diagnostic and Statistical Manual of Mental Disorders (DSM)-5 criteria. The MINI is a 30-min fully structured interview that was designed to be administered in a fraction of the time compared with other interviews which allows an assessment of 17 of the most common mental health disorders.^54 55^ To reduce the burden on patients from an excessively long interview before surgery, we will only collect the information necessary to establish diagnoses of major depression and generalised anxiety disorder. The exclusion criteria of acute suicidality will also be assessed using a specific item from the MINI. The interview was conducted in the context of evaluating major depression. In the case of acute suicidality, medical staff will be informed, and patients will be referred to appropriate mental health counselling services. In addition, study staff will ask patients about any previous mental disorder diagnoses made by a health professional, as well as prior mental health treatments, including psychotherapy and antidepressant use. Additionally, post-traumatic stress disorder (PTSD) screening will also be conducted using three items from the Structured Clinical Interview for DSM-5.^56^

##### Depressive symptoms

The PHQ-9 will be used to assess the severity of self-rated symptoms of depression over the last 2 weeks.^57^ In contrast to more extensive questionnaires like the Beck Depression Inventory, the PHQ-9 refers to the nine DSM-5 criteria for major depression which are anhedonia, depressed mood, sleep problems, feelings of tiredness, changes in appetite, feelings of guilt or worthlessness, trouble concentrating, feeling ‘slowed down’ or restless and suicidal ideation. Individual symptoms were assessed using a 4-point Likert scale ranging from 0=‘not at all’ to 3=‘nearly every day’. An overall score ranging from 0 to 27 for the PHQ-9 was calculated by summing the individual items. Scores of 0–4, 5–9, 10–14, 15–19 and 20–27 indicate minimal, mild, moderate, severe and very severe symptom severity, respectively. The standard cut-off score of 10 corresponds to a diagnosis of a depressive episode with a sensitivity and specificity of 88% each.^55^ A recent individual participant data meta-analysis yielded a sensitivity of 77% and a specificity of 88% for this cut-off compared with the MINI diagnostic interview as the reference standard for a diagnosis of a major depressive episode.^55^

A science advisory from the American Heart Association, endorsed by the American Psychiatric Association, recommended the PHQ-9 as a brief depression screening instrument with reasonable sensitivity and specificity for patients with CHD, which most patients are able to complete without assistance in 5 min or less.^58^ Moreover, irrespective of a formal diagnosis of a major depressive episode, a score of 10 or higher on the PHQ-9 is capable of stratifying patients with CHD in terms of an increased risk of subsequent cardiovascular events, allowing clinicians to prioritise and personalise treatment to improve outcomes.^59^ The PHQ-9 is an economical and valid instrument for assessing depressive symptoms in patients who have undergone cardiac surgery, including those awaiting CABG.^60^ The PHQ-9 has been used in numerous studies on depression in patients with CABG, including for the assessment of depressive symptoms before, 2–6 months after and 1 year after surgery. Meta-analytic data revealed a prevalence of depression of 22% (95% CI 12% to 33%) before and of 18% (95% CI 14% to 23%) after CABG surgery when assessed using the PHQ-9.^7^

We will use two complementary approaches for the analysis, correcting for multiple tests when assessing the significance of the accuracy of the prediction model. One approach frames the prediction challenge as a binary classification problem, using a PHQ-9 cut-off score ≥10 for defining the presence versus absence of CSDSs. The second approach views the prediction challenge as a regression problem and tries to predict individual PHQ-9 scores without applying any threshold.

##### Anxiety symptoms

The GAD7 questionnaire (exploratory outcome) will be used to measure the severity of self-rated anxiety symptoms in the last 2 weeks.^61^ Seven items are rated on a 4-point Likert scale ranging from 0=‘not at all’ to 3=‘nearly every day’, yielding a total score between 0 and 21. Sum scores of 10 points or higher indicate a moderate to severe level of anxiety symptoms (ie, clinically significant symptoms), corresponding to a diagnosis of a GAD with a sensitivity of 89% and a specificity of 82%.

##### Post-traumatic stress

The PCL-5 will be used to assess the severity of post-traumatic stress induced by CABG. The PCL-5 is a 20-item self-report measure that assesses the 20 DSM-5 symptoms of PTSD in the past month referring to intrusions (eg, repeated, disturbing and unwanted memories of the cardiac surgery and hospital stay), avoidance, negative alterations in cognition and mood, and alterations in arousal and reactivity. Each item is rated on a 5-point Likert scale ranging from 0 (‘Not at all’) to 4 (‘Extremely’). Sum scores of 33 or higher indicate probable PTSD.^62^

##### Health-related QoL

The SF-12 will be used to assess physical and mental health-related QoL, asking about physical functioning, role limitation due to physical health problems, bodily pain, general health, vitality (energy/fatigue), social functioning, role limitation due to emotional problems and mental health (psychological distress and psychological well-being) in the last 4 weeks.^63^ In patients who underwent CABG, the SF-12 is a valid QoL measure with an optimal balance between brevity and psychometric validity and sensitivity.^64^ Compared with the SF-36, the SF-12 was shortened by two-thirds in length. In patients with CHD, the mental and physical summary measures replicate the SF-36 summary measures well and can adequately replace them.^65^

##### Additional questionnaire-based psychometric measures

To assess self-rated health, we will present the single-item question ‘In general, would you say your health is excellent, very good, good, fair or poor?’ along with the SF-12 survey.^66^ Social support will be measured using the Enhancing Recovery in Coronary Heart Disease Patients Social Support Inventory, which assesses the availability of support from any network member using six items. These refer to emotional, practical and informational support. Each item is rated on a 5-point Likert scale from 1 (‘None of the time’) to 5 (‘All of the time’), yielding a sum score between 6 and 30.^67^ Patients’ beliefs (cognitive and emotional representations, and illness comprehension) of their cardiac disease before surgery^68^ will be assessed using the brief German version of the self-rated Brief Illness Perception Questionnaire.^69^ Each of eight items covering the dimensions ‘consequences’, ‘time-line’, ‘personal control’, ‘treatment control’, ‘identity’, ‘illness concern’, ‘coherence’ and ‘emotional representation’ is scored between 0 and 10, yielding a sum score between 0 and 80. The term ‘illness’ will be modified to ‘heart disease’, as previously described.^70^ Two variables from positive psychology, purpose in life (assessed using the Purpose in Life Questionnaire)^71^ and optimism (assessed using the Revised Life Orientation Test)^72^ are additionally assessed. The Nordic Patient Experiences Questionnaire, a short survey on the Patient Reported Experience Measure, is also collected.^73^

### Inflammation markers

The circulating levels of hsCRP, IL-6, TNF-α, IL-1β, IFN-γ, fibrinogen and D-dimer will be assessed at two time points: pre and post perioperative time points. Following strict in-house protocols to ensure optimal pre-analytical conditions, morning blood samples (fasting, if possible) of 10 mL will be collected and processed into plasma samples 1 day before surgery (baseline) to assess low-grade systemic chronic inflammation. To assess the acute inflammatory response, blood will be sampled on the second postoperative day when CRP peak values are expected after uncomplicated CABG surgery.^74^ Again, blood samples of 10 mL will be processed into plasma samples. While the processing of blood samples from USZ patients takes place in the Stress and Behavioral Research Laboratory of the USZ PSY at the University Hospital Zurich, the processing at the Stadtspital Zurich Triemli takes place on site after blood collection using a mobile centrifuge. The plasma aliquots are then transported from Triemli Hospital to the University Hospital Zurich on crushed ice by the study staff of USZ using a standardised procedure. The procedure is recorded in writing. All plasma aliquots will then be stored at the University Hospital Zurich at −80°C until measurement using high-sensitivity assays in the Stress and Behavioral Research Laboratory of the University Hospital Zurich, following standard procedures. The principal inflammatory measure hsCRP will be quantified in plasma using a high-sensitivity latex-enhanced immunoturbidimetric assay on a Cobas c 501 autoanalyzer (Roche Diagnostics, Mannheim, Germany).^75^ Fibrinogen will be measured using the Clauss method and D-dimer using a quantitative sandwich enzyme immunoassay (IBL Hamburg, Germany; RUWAG, Bettlach, Switzerland).^76^

### Assessment of cardiac interoception and related measures

We will apply the HBCT as a physiological marker of cardiac interoception (cardioception).^72 77^ The task requests from participants to pay attention to their own heart sensations in a sitting position (with both feet on the floor and hands on the thighs). They must count their heartbeats within three intervals in random order and verbally state the number. Each interval begins with a verbal start signal and ends with a stop signal by the experimenter. Participants are assured that zero is a valid response if they do not feel a heartbeat. But they are also encouraged to count the heartbeat even if they feel only a slight sensation. The actual task is preceded by a 15 s practice period. The subjects are also given no information about the duration of the task or their performance. The actual heartbeat is recorded with an ECG. The ambulatory ECG device is provided by USZ. The heartbeat perception score will be calculated as the average (over the three intervals) of (1−(|Heart Beat recorded−Heart Beat counted|)/Heart Beat recorded). In our study, we also consider scores below 0 (down to −1), which can occur when participants count substantially more heartbeats than were actually recorded. This approach allows us to capture the full range of heartbeat perception performance, including extreme overestimation. Low scores in the heartbeat perception are associated with low cardiac perception, while high scores are associated with high cardiac perception. The HBCT is still used in clinical settings despite criticism of its validity because it offers a simple and non-invasive way to assess interoceptive abilities. In particular, the HBCT provides a quick evaluation of a person’s ability to perceive internal bodily signals, which is relevant for diagnosing and treating psychosomatic and affective disorders. Additionally, it is easy to standardise and cost-effective to administer, making it a practical tool despite its methodological limitations. Especially for patients admitted to the clinic just 1 day before surgery, who undergo many other examinations, the HBCT offers a feasible approach to approximate interoceptive ability without adding a significant burden.

We will apply version 2 of the Multidimensional Assessment of Interoceptive Awareness (MAIA-2) self-report questionnaire,^78^ an improved version of the original MAIA, to assess two dimensions of interoceptive awareness, which are ‘Not-Worrying’ (five items; eg, ‘I can notice an unpleasant body sensation without worrying about it’) and ‘Trust’ (three items; eg, ‘I trust my body sensations’). Each item is rated on a 6-point Likert scale ranging from 0 (never) to 5 (always).

Patients will complete the physical symptom and mood assessments. Physical symptom assessment was based on the Somatic Symptom Scale (SSS-8)^79^ and cardiac items from the Anxiety Sensitivity Index-Revised (ASI-R).^80^ Each of the eight items of the SSS-8 is rated on a 5-point Likert scale from 0 (not at all bothered) to 4 (very much bothered) yielding a total severity score of bodily distress between 0 and 32. Responses to the four items of the ASI-R (eg, ‘it scares me when my heart beats rapidly’) are scored on a scale from 0 (very little) to 4 (very much) yielding a total score of ongoing cardiac threat perception between 0 and 16. The Global Mood Scale to measure negative and positive moods with 10 items each, yielding total negative and positive mood scores.^81^ heart rate variability (HRV) (the root mean square of successive differences between consecutive heartbeats (RMSSD)) will also be assessed with ECG. The ECG data for calculating HRV are collected the day before surgery or the data will be taken from the patient charts. If necessary, suitable software and codes will be used for the evaluation of the data.

### Statistical analysis

All analyses using ML will be conducted at the Institute for Biomedical Engineering, Translational Neuromodeling Unit (TNU), Swiss Federal Institute of Technology Zurich. In addition to the ML analyses, we plan to perform supplementary analyses that are restricted to the training set only (to avoid information leakage and bias in prediction). These additional analyses will include parametric and non-parametric tests (depending on data distribution) for group comparisons, correlation analyses and multivariable or multivariate linear and logistic modelling.

Concerning our clinical outcome of main interest (post-CABG CSDSs), two predictive models will be developed: (1) a binary classification model to predict presence versus absence of CSDSs (defined as PHQ-9 score ≥10) and (2) a regression model to predict the continuous PHQ-9 score 6 weeks postoperatively. Model development and internal validation will be performed using the training dataset (n=200, data acquired at the University Hospital Zurich), while external validation will use an independent test set (n=100, data acquired at the Stadtspital Zurich Triemli). The two datasets will remain strictly separated throughout data collection and analysis in order to avoid information leakage.

Internal validation will use nested cross-validation combined with repeated random subsampling (also known as Monte Carlo cross-validation).^82^ Hyperparameter tuning will occur in a 10-fold inner loop, and model selection in a 10-fold outer loop. The procedure of training and selecting models will be repeated 100 times, each time randomly dividing the training dataset into folds. Our approach is designed to fulfil a dual purpose: protecting against overfitting (nested cross-validation) and obtaining stable and accurate estimates of predictive performance (repeated random subsampling).

For classification, we will explore multiple algorithms, including logistic regression (with elastic net regularisation), random forest, gradient boosting, k-nearest neighbours, support vector machines, neural networks and naïve Bayes. For regression, elastic net regression will be used, balancing L1 and L2 penalties to optimise variable selection and minimise overfitting.^83^ In the outer loop, models will be selected based on their performance. For classification, the key performance metric for model selection will be balanced accuracy. Additionally, we will also compute sensitivity, specificity, positive predictive value (PPV), negative predictive value (NPV) and area under the receiver operating characteristic curve (AUROC) of the winning model. For regression, metrics such as root mean square error (RMSE) and R^2^ will be reported, with model selection based on the lowest RMSE. External validation will apply the model that was selected for the training set to the test dataset, using the same metrics and permutation tests. SHapley Additive exPlanations (SHAP) values will be used to interpret predictor importance and enhance explainability.^84^ The statistical significance of predictions will be assessed via permutation testing.

### Pre-registration of analyses and code review

Before data are accessed for the purpose of model training, a detailed analysis plan will be preregistered. Once training is completed, the choice of the selected models and the procedure for model validation on the held-out test set will be preregistered as well, in an amendment to the analysis plan. Prior to submitting the results for publication, the analysis code will be peer-reviewed, following established practices at the TNU, Zurich.

### Patient and public involvement

Patients and members of the public were not involved in the design, conduct, recruitment or outcome selection for this study. The research questions and methodology were developed by the clinical and research team based on current gaps in the literature and clinical relevance. While patient input was not included in planning or dissemination phases, future efforts will aim to incorporate patient perspectives in the development and communication of research findings.

## Ethics and dissemination

The responsible ethics committee has reviewed and approved this project (Kantonale Ethikkommission Zürich, BASEC number: 2023-02040). This study is conducted by the principal investigators at both study centres: RvK (Department of Consultation-Liaison Psychiatry and Psychosomatic Medicine, University Hospital Zurich, USZ) and OD (Department of Cardiac Surgery, USZ and Municipal Hospital of Zurich Triemli). The study protocol has been reviewed and approved in accordance with Swiss legal requirements, the Declaration of Helsinki and institutional standards for research integrity involving human participants. All participants will provide written informed consent prior to enrolment.

Although data collection adds some burden, efforts have been made to minimise it by aligning assessments with routine care, using brief validated instruments and limiting the scope of psychiatric interviews to depression and anxiety.

Ethical safeguards include deferring disclosure of psychiatric diagnoses until the 6-week follow-up to avoid presurgical distress. Support will be offered if CSDSs or anxiety persists. Participants are informed of their rights, including the option to withdraw consent and how their data and biological material will be handled. The study respects participant autonomy while prioritising clinical relevance and feasibility.

Results from this study will be shared through peer-reviewed publications, conference presentations and stakeholder meetings involving clinicians and mental health professionals. In addition, findings will be communicated to participating centres and patient communities in accessible formats, ensuring that knowledge gained can inform both clinical practice and future research.

## Discussion

The aim of this study is to develop and validate prognostic models to predict CSDSs and other psychological outcomes, including anxiety, post-traumatic stress symptoms and QoL, 6 weeks after elective CABG surgery. Psychological morbidity, particularly CSDSs, remains a prevalent yet under-addressed complication following CABG. Despite growing recognition of its impact on recovery and QoL, robust tools for predicting psychological risk after surgery are currently lacking. This study seeks to fill this critical gap by developing and validating predictive models that incorporate a broad range of factors including demographic, clinical, psychometric, inflammatory and interoceptive markers to forecast the development of CSDSs and other psychological outcomes post-CABG. Importantly, this protocol adopts a prospective, multicentre design and applies state-of-the-art ML methods for model development and validation. The inclusion of biological correlates, such as hsCRP, IL-6, TNF-α and cardiac interoceptive awareness, offers a novel, multidimensional approach to risk stratification. These variables have shown preliminary associations with depression in cardiac populations, but their predictive value within an integrated prognostic framework remains to be determined.

The inclusion of both categorical and continuous assessments of depressive symptoms using the PHQ-9 will provide a differentiated view of the outcomes and increase clinical applicability. Moreover, the development of a simplified ‘light solution’ model aims to support real-world use in busy clinical settings, thereby making stratified care pathways more feasible.

Collectively, by providing an evidence-based foundation for early identification of patients at risk, this study has the potential to shift the paradigm in perioperative care from reactive to proactive. If validated, the resulting models could inform tailored interventions, guide resource allocation and ultimately improve psychological and functional recovery after CABG.

This project aims to generate robust, novel insights into predictors of postsurgery CSDSs in CABG patients, focusing on inflammation and interoception. These findings may support precision medicine by identifying at-risk individuals and guiding early interventions, which could include psychosocial support, behaviour change strategies or improvements in interoception potentially integrated into existing cardiac rehabilitation programmes.

Given that one in four patients undergoing CABG experiences CSDSs, our predictive model could facilitate early identification and monitoring, improving QoL and long-term outcomes.

While this study aims to develop robust predictive models for psychological outcomes post-CABG, several limitations should be acknowledged. First, the observational design precludes causal inference regarding identified predictors. Second, although the study uses a multicentre approach, findings may not be generalisable beyond the Swiss healthcare context or to patients undergoing emergency CABG or those with significant comorbidities excluded from the sample. Third, while ML techniques enhance predictive accuracy, they also risk overfitting, particularly in relatively small datasets; careful model validation and transparency in reporting will be essential. Fourth, reliance on self-report measures for psychological outcomes, though validated, may introduce reporting biases. Lastly, although the study includes interoceptive and inflammatory biomarkers, other potentially relevant biological or psychosocial factors may not be fully captured, and the feasibility of implementing the ‘light solution’ model in routine clinical care will need further real-world testing.

Collectively, this study has the potential to significantly improve perioperative mental healthcare by enabling early identification of patients at risk for CSDSs and related psychological outcomes after CABG. By integrating demographic, clinical, biological and psychometric data into predictive models, it supports a more proactive, personalised approach to care. While further validation and real-world implementation will be necessary, the findings may ultimately inform targeted interventions and enhance recovery and QoL in this vulnerable patient population.
