# Supplementary material for: Predictive modelling of clinically significant depressive symptoms after coronary artery bypass graft surgery: protocol for a multicentre observational study in two Swiss hospitals (the PsyCor study)
Source: BMJ Open. 2025 Sep 5;15(9):e108061. doi: 10.1136/bmjopen-2025-108061 (PMC12414220; doi:10.1136/bmjopen-2025-108061)
Supplement: Supplementary file 1 [file bmjopen-2025-108061.reviewer_comments.pdf]

PEER REVIEW HISTORY

BMJ Open publishes all reviews undertaken for accepted manuscripts. Reviewers are asked to complete a checklist review form and are provided with free text boxes to elaborate on their assessment. These free text comments are reproduced below.

ARTICLE DETAILS

Title (Provisional)

Predictive modelling of clinically significant depressive symptoms after coronary artery bypass graft surgery: protocol for a multicenter observational study in two Swiss hospitals (the PsyCor study)

Authors

Lazaridou, Asimina; Sivakumar, Sinthujan; Rodriquez, Hector Cetina; Weilenmann, Sonja; Princip, Mary; Hackl-Zuccarella, Claudia; Petzschner, Frederike; Jakob, Heinzle; Stephan, Klaas; Dzemali, Omer; von Känel, Roland

---

This article was not externally reviewed at BMJ Open. Protocols that have been independently assessed prior to submission to BMJ Open are usually fast-tracked to publication on the grounds that further substantial changes will not be possible. This independent assessment will usually be external, independent review for both a substantial grant award from a non-commercial or government funder and ethics approval.
